# Barriers of access to care in a managed competition model: lessons from Colombia

**DOI:** 10.1186/1472-6963-10-297

**Published:** 2010-10-29

**Authors:** Ingrid Vargas, María Luisa Vázquez, Amparo Susana Mogollón-Pérez, Jean-Pierre Unger

**Affiliations:** 1 Health Policy and Health Services Research Group, Health Policy Research Unit, Consortium for Health Care and Social Services of Catalonia, Av. Tibidabo 21, Barcelona 08022, Spain; 2 Escuela de Medicina y Ciencias de Salud, Universidad del Rosario, Carrera 24 n° 63 C - 69, Bogotá D.C. 11001, Colombia; 3 Department of Public Health, Prince Leopold Institute of Tropical Medicine, Nationalestraat 155, Antwerp B-2000, Belgium

## Abstract

**Background:**

The health sector reform in Colombia, initiated by Law 100 (1993) that introduced a managed competition model, is generally presented as a successful experience of improving access to care through a health insurance regulated market. The study's objective is to improve our understanding of the factors influencing access to the continuum of care in the Colombian managed competition model, from the social actors' point of view.

**Methods:**

An exploratory, descriptive-interpretative qualitative study was carried out, based on case studies of four healthcare networks in rural and urban areas. Individual semi-structured interviews were conducted to a three stage theoretical sample: I) cases, II) providers and III) informants: insured and uninsured users (35), health professionals (51), administrative personnel (20), and providers' (18) and insurers' (10) managers. Narrative content analysis was conducted; segmented by cases, informant's groups and themes.

**Results:**

Access, particularly to secondary care, is perceived as complex due to four groups of obstacles with synergetic effects: segmented insurance design with insufficient services covered; insurers' managed care and purchasing mechanisms; providers' networks structural and organizational limitations; and, poor living conditions. Insurers' and providers' values based on economic profit permeate all factors. Variations became apparent between the two geographical areas and insurance schemes. In the urban areas barriers related to market functioning predominate, whereas in the rural areas structural deficiencies in health services are linked to insufficient public funding. While financial obstacles are dominant in the subsidized regime, in the contributory scheme supply shortage prevails, related to insufficient private investment.

**Conclusions:**

The results show how in the Colombian healthcare system structural and organizational barriers to care access, that are common in developing countries, are widened by both the insurers' use of mechanisms that limit the utilization and the public healthcare providers' change of behavior in a competition environment. They provide evidence to question the promotion of the managed competition model in low and middle-income countries.

## Background

The managed competition model is one of the reforms promoted in the last few decades in Latin America, in response to the objective to improve equity and efficiency of health systems. It has been characterized by the introduction of a regulated market for health insurance to correct market failures [1]. Under managed competition, insurance companies are responsible for providing or arranging the provision of health services for their enrolled members, through their own providers or through contracted ones. The Colombian experience is considered to be one of the first examples of implementing managed competition in a low-income country [2].

### The managed competition model in Colombia

**Figure 1:**
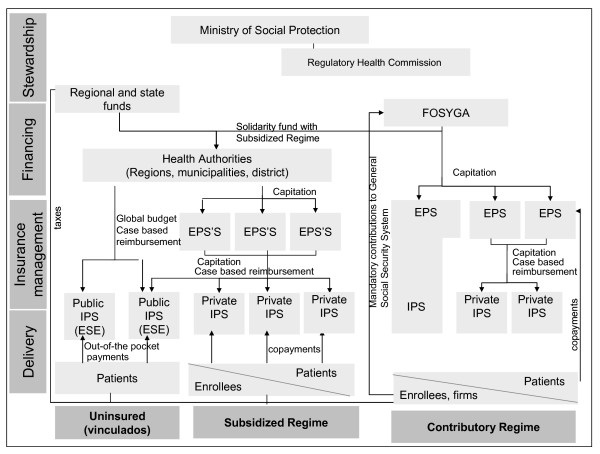
**The model of managed competition in the Colombian healthcare system**. Figure legend text: FOSYGA: Fondo de Solidaridad y Garantía (Solidarity and Guarantee Fund); EPS: Empresa Promotora de Salud (Insurance Company for the Contributory Regime); EPS'S: (Insurance Company for the Subsidized Regime); IPS: Instituciones Prestadoras de Servicios de Salud (Healthcare Provider); ESE: Empresa Social del Estado (Public Health Provider). → Monetary flows. Source: authors.

Colombia radically reformed its healthcare system with Law 100 of 1993 [3], which created the General Social Security System in Health, with two insurance schemes: the Contributory Regime for formal sector employees and people able to pay, financed by mandatory contributions [3]; and the Subsidized Regime for people without the ability to pay, funded by resources from the Contributory Scheme and other sources of financing, such as taxes (Figure 1). Healthcare Insurers (Empresas Promotoras de Salud - EPS) were introduced for managing the Contributory Regime, as well as Subsidized Regimen (Empresas Promotoras de Salud Subsidiadas - EPS'S). They were to compete for the enrolment of population and received a capitation payment to cover different benefit packages in each regime (Plan Obligatorio de Salud - POS and Plan Obligatorio de Salud Subsidiado - POS-S) [4]. Currently, the contributory market is characterized by the predominance of private insurers - 86.1% of the affiliation - and the concentration in 5 private insurers that hold 50% of markets share [5]. The largest public insurer has been transformed into a mixed company with private capital and 5.8% of membership [5]. Competition for contracts with the insurers was also introduced among public and private healthcare providers (Instituciones Prestadoras de Salud - IPS). Healthcare for the uninsured (*vinculados*) and services excluded from the POS-S are provided by public hospitals funded by local and regional authorities [6], that represent 31.3% of total healthcare providers [7]. The uninsured have to pay for services and the insured make a co-payment according to their income [8].

The reform of the Colombian healthcare system has been, and still is, presented as a successful experiment in improving access to care [9,10]. However, it has been a long, complicated process, and the results are controversial [11,12]. In spite of the significant increase in public health expenditure from 3% to 6.6% of GDP, over the 1993 to 2007 period [13], around 15.3% to 19.3% of the population remains uninsured [14,15]; and 38.7% are insured under the subsidized regime [15] that covers a range of services (POS-S) greatly inferior to that provided by the contributory one [16,17]. Approximately 17% of health expenditure is devoted to administrative costs [18], of which more than 50% is spent on supporting daily operations (financial, personnel, and information management) and enrollment processes [19].

Furthermore, several studies seem to indicate a decrease in realized access to services [20,21], and point to significant barriers related to characteristics of population, such as insurance enrolment [22-28], income [22,25,26,28], education [22-27,29] and, characteristics of services, such as geographic accessibility and quality of care [26,30]. In 2005, the maternal mortality rate, an indicator that is sensitive to the overall healthcare system, was 130/100.000 in Colombia, compared to 30/100.000 in Costa Rica, while per capita 2004 health expenditure were similar (USD 549 and USD 598, respectively) but a GNP per capita lower in the former (USD 6130 and USD 9220) [31].

In addition, available evidence points to failures in the *condition sine qua non *for the successful implementation of managed competition, according to its supporters [1]: the existence of an effective regulatory system. These studies [32-35] reveal deficiencies in regulation authorities in their ability to control a great number of institutions related to insufficient financial resources, lack of control mechanisms and excessive, and sometimes contradictory, regulation norms.

Most studies of the determinants of use of care in Colombia concentrate on personal variables and initial contact with services, and ignore contextual variables - health policy and characteristics of healthcare services. Insurance coverage, measured only by enrolment rate, is often viewed as an independent variable, although in managed competition models, insurers directly influence the provider networks and conditions of access to healthcare [36]. In addition, little research has evaluated access from the point of view of the social actors [26,37-39], despite the limited capacity of quantitative models in explaining determinants of use of care, due to methodological difficulties in including contextual variables [40,41].

The objective of this article is to contribute to the improvement of our understanding of the factors influencing access to the continuum of healthcare services in the Colombian managed competition model, from the perspective of social actors.

## Methods

There were two **Areas of Study**: one urban (Ciudad Bolívar, Bogotá, D.C.) and one rural (La Cumbre, Department of Valle del Cauca) with 628.672 [42] and 11.122 inhabitants [43] respectively. In the former, a wide array of insurers are present, while in the latter only one subsidized insurance company, with the majority of the contributory insurance enrollees being affiliated in two insurance companies. In both areas most of the population live in poverty [42]. In the urban area, the coverage of the subsidized regime is slightly less than in the rural area (30% compared to 37.5%), whereas the percentage of contributory regime enrollees is markedly higher (43.7% compared to 9.7%) [42,44]. The rest remain uninsured (26.3% and 47.2% respectively).

The **Study Design **was qualitative, exploratory, and descriptive-interpretative, based on a case study of four healthcare service networks. The case study provides extensive information about the phenomenon - access to care in the managed competition model in Colombia - based on individual cases [45,46]. The analysis of access to care is based on the theoretical frameworks of Aday and Andersen [47], and Gold [36]. The first distinguishes factors that influence access to care related to health policies, the characteristics of healthcare services - resources and organization - and the population. Under organizational determinants of access, Aday & Andersen's framework refers, among others, to the manner in which medical personnel and facilities are coordinated and controlled in the process of providing health services [47]. Gold's theoretical model adapts the former to incorporate factors related to insurance companies, to better understand how organizational structures developed by *managed care *models affect access. This framework acknowledges the influence of managed care organizations on the way individuals are covered by their health plans by defining the provider network and the way in which patients access it [36].

In this study, access to care is considered to be the use of services along the continuum of care [48].

A theoretical **Sample **was selected in three stages. I) Case studies: healthcare networks of both insurance regimes, in both the rural and urban areas. The network is defined as the insurer and their healthcare providers, either contracted and/or owned; II) Public and private providers from different levels of care, and with different ownership relationship to the insurance company; III) Informants, searching for variation in discourse: a) insured and uninsured users, over the age of 18, who would have used or tried to use at least two levels of care in the past six months; b) health and administrative professionals with at least one year of experience; and c) managers of the providers and insurers. Users were selected through the first level of care records and, when not available, with help of the health professionals who provided their care. For the others, an institutional contact identified possible informants according to the criteria above. Informants were then contacted and invited to participate. The final sample size, between 24 and 61 informants per case study, was reached by saturation of the information (Table 1). The differences are due to uninsured people being incorporated as informants into the subsidized networks, and because rural EPS-S managers and administrative professionals refused to participate.

**Table 1:** Final composition of the informants' sample

Category of key actors		Urban area		Rural area	
		
		Subsidized network	Contributory network	Subsidized network	Contributory network
**Healthcare users**	Insured	12	6	9	8

	Uninsured	6	0^(*)^	6	0^(*)^

**Healthcare professionals**	Firs level of care	10	4	7	7
	
	Secondary and tertiary level of care	11	4	4	4

**Administrative personnel**	Providers	4	1	4	3
	
	Insurers	6	2	0^(**)^	0^(***)^

**Managers**	Providers	6	4	4	4
	
	Insurers	6	3	0^(**)^	1

Total		61	24	34	27

^(*) ^The network only provides care to insured healthcare users

^(**) ^The Subsidized Regime Insurer (EPS'S), the only one in the area, refused to participate in the study

^(***) ^The contacts of this category refused to participate due to problems with their agendas

### Data collection

Individual semi-structured interviews with a topic guide were conducted. The guides - with a common section and a specific section for each informant group - included opinions on access to the continuum of services across the network, elements that influence it, and strategies for improvement. In the user guide, users' experience with services was also explored. Interviews with users were conducted at their homes, the remainder of informants mostly in the workplace. The interviews lasted between one and two hours and were audio-recorded and fully transcribed.

### Data analysis and quality of information

A content analysis [49] was conducted with the support of the software Atlas-ti 5.0. The process of generating categories was mainly inductive. Data were segmented by case study, informant's group, and themes. Themes were identified, coded, re-coded and classified identifying common patterns by looking at regularities, convergences and divergences in data, through a process of constant comparisons, going back and forth between data and conceptual framework. In order to ensure the quality of results, the information was triangulated between groups of informants and the results were contrasted with the informants and the bibliography. In addition, four researchers with different backgrounds and in-depth knowledge of the context participated in the analysis.

### Ethical considerations

Participants were informed of the objective of the study and that they were free to participate and to leave at any point. Participants gave oral consent for their participation and the interviews to be recorded. The recordings and transcripts were codified in such a way that the individual origin of each one could not be identified, before being appropriately stored. These results are part of a broader research aimed at analyzing the impact of integrated healthcare networks on health care access and efficiency, in Colombia and Brazil. Ethical approval was sought and obtained from the University of Rosario's Ethical Committee in 2005.

## Results

The general perception is that access to healthcare along the continuum of care is, in general, complex and does not suit population needs. Difficulties especially arise in access to specialized care, although serious problems were also identified in access to the first level of care in the contributory regime in the urban area. Four groups of interrelated obstacles emerged: the insurance design, insurers' managed care and purchasing mechanisms, the characteristics of the network of healthcare providers and, to a lesser extent, the socio-economic characteristics of the population. Competition in insurance management and in the provision of care emerges as a barrier that influences all others. In the informant's discourses, important differences emerged in the intensity of barriers depending on the area and insurance scheme (Table 2).

**Table 2:** Difficulties in access to healthcare by type of barrier, insurance regime and area

	Subsidized Regime		Contributory Regime	
	
	Urban area	Rural area	Urban area	Rural area
**Insurance design**	POS-S low coverage of specialized care	POS-S low coverage of specialized care	Norms limiting access to medical care	Norms limiting access to medical care
	
	Classification of services by level of care	Classification of services by level of care		
	
	Co-payments for non-POS-S services	Co-payments for non-POS-S services (chronic and high-cost illnesses)		
	
	Out-of-pocket (basic specialized care)	Out-of-pocket (basic specialized care)		

**Insurance companies**	Maximization of benefits			
	
	Managed care mechanisms	Managed care mechanisms	Managed care mechanisms	Managed care mechanisms
	- authorizations	- authorization requirements	- limits to clinical practice	- limits to clinical practice
	- capitation payment			- authorization requirements
	
	Conflict in the interpretation of services included in the POS-S			
	
	Purchase of services			
	- fragmented contracting			
	- change in contracted providers			

**Network of healthcare providers**	Public healthcare providers' search for economic profit	Public healthcare providers' search for economic profit		
	
	Shortage of basic and high technology specialized care	Shortage of basic and high technology specialized care	Shortage of basic specialized care and primary care	
	
		Distance to primary and specialized care	Distance to primary and specialized care	Distance to specialized care
	
	Waiting time for specialized care	Waiting time for specialized care	Waiting time for specialized care	Waiting time for specialized care
	
	In-person and restricted appointment requirements	In-person and restricted appointment requirements		
	
	Poor quality of care			Poor quality of care

**Population characteristics**	Low income level	Low income level	Low income level	Low income level
	
		Lack of family support	Lack of family support	Lack of family support

POS-S: Subsidized Regimen Benefit Package

Source: authors

### Barriers related to the insurance design

The informants of the subsidized regime, in both areas, reported difficulties of access to healthcare related to the insurance design: exclusion from benefits of necessary specialized services, classification of services by levels of care and co-payment (Table 3). The informants, especially secondary care specialists, considered that blocked access to tests classified as lower level in the more complex healthcare levels, as well as the inclusion of only very basic diagnostic tests in the first level, force patients to go back and forth between several health facilities and delay the resolution of the health problem.

**Table 3:** Examples of quotations regarding "barriers related to the insurance design"

Category	Quotations
Low coverage of specialized care of the subsidized benefits package	*"the subsidized regime is very limited, so it has a benefit plan that covers certain diseases and procedures. There is a great number of diseases, drugs and exams that are not covered (...)" *(manager, public specialized care provider - subsidized rural network)

Classification of services by level of care	*"the fragmentation has been very serious, and very harmful, (...) here they fragmented the diseases (...). I mean, a patient can't get pneumonia and fungus on his feet because we treat pneumonia here, but fungus is treated in another place, so no, we can't treat any diseases here that aren't level III of care" *(health professional, public secondary care provider - urban subsidized network)

Conditions that restrict access to services	*"you have 70 weeks of coverage and they have to pay 30% of 100 million pesos [33.334€], where are they going to get it?*" (administrative professional, private secondary care provider - rural contributory network)

Finally, out-of-the-pocket payments - or co-payment for the insured - emerge as one of the biggest barriers. Enrollees of the subsidized regime point to co-payment of services not covered - specialized diagnostic tests and medicines for chronic and high-cost diseases - while the uninsured also mention out-of-the-pocket payment for basic specialized services as a barrier. When confronted with the economic barrier, the informants often desisted in seeking medical care, "*... they ordered me those for the thyroid, and there were some exams I need to do that cost about three million [EUR 1.000] (...) the doctor said, 'Well, sell your house or whatever you have', and I said, 'What house? I got the TSH because that is covered, but not the others, because they're too expensive*" (user, subsidized regime - rural area). In the discourse of the contributory regime's informants, there strongly emerge norms limiting access to medical care - minimum contributory's period for high cost services or suspension of insurance coverage for lack of payment. As a consequence, they cannot access health services or end up having to make a co-payment (Table 3).

### Barriers related to insurers

The presence of the insurance companies in the healthcare system emerges, in the interviews with managers and professionals of public facilities, as a barrier to access for those who are insured under the subsidized regime (Table 4). On the one hand, insurers try to maximize their benefits by introducing mechanisms to reduce the use of services. On the other, its presence requires re-routing funds from medical care to intermediation: "*all these intermediaries we have earn money based on how much they can avoid directing it to hospitals, so they end up keeping the biggest piece of the pie*" (health professional, public primary care provider - urban subsidized network).

**Table 4:** Examples of quotations regarding "barriers related to insurers"

Category	Quotations
Introduction of intermediaries that maximize benefits	"*Intermediation is harming the provision of services. Health enters the market and that's when all the costs and quality problems start, which modify all of the activities. So the financial event becomes more important than the health one*" (Manager, public secondary care provider - urban subsidized network)

Use of managed care mechanisms for cost reduction	*"the auditors and those responsible for authorizations in the insurance companies, ... their job shouldn't be to try to stop authorizations, as they do now, basically because of costs" *(health professional, private secondary care provider-rural subsidized network)

Authorization	*"(...) they should give us the order, and that's all. You see, they send [me] over to the insurance company, and they'd say no, that I had to bring the others [doctor's orders]. I had to go to where he was hospitalized (...) They have you running all around (...) And run. And it [the money] disappears in a flash, you hear, in bus tickets and everything else. So we don't have all that money to run around...bus tickets and the rest" *(user, subsidized regime - rural area)

Capitation payment	*"(...) the first level is capitated, and that is a very perverse contract mechanism. In a poor system, in a poor country, because you have to sacrifice quality. So health professionals are pressured to do the minimum, the minimum, because the cost is fixed, and if they go beyond the minimum, then the contract is no longer worth it, it's not profitable anymore. So quality is often sacrificed in this contract system*" (health professional, private secondary care provider - urban subsidized network)

Conflict in interpretation of health services included in the subsidized benefits package (POS-S)	*"the insurance company and the municipal health secretariat start throwing the ball back and forth in an incredible way. Both start to create strategies so that the other will have to provide care for the patient...(...) So the poor patient ends up being thrown from one side to the other until finally he dies or he gets added complications" *(manager, private secondary care provider - urban subsidized network)

Fragmented contracting	*"the ARS *[previous name for subsidized regime insurance company] *owns the patient... So it contracts this hospital for this, the other (hospital) for that... so I do a piece here, another there, another there (...)" *(health professional, public secondary care provider - urban subsidized network)

Better access to the continuum of care for the uninsured	*"(...) I pick up the list and if I need a specific specialty, I look for where it is for "vinculados" *[the uninsured], *where the waiting time is shorter and I send him there (...) That part lets one play with the windows of waiting list. In the Subsidized regime, you don't have this waiting time, because you're limited to what the insurers have contracted" *(manager, primary care provider - urban subsidized network)

POS-S: Subsidized benefit package

The informants from both insurance regimes identify the managed care mechanisms employed by insurers as a barrier to use of services (Table 4). The mechanisms they identified differ according to the healthcare network type. In networks where there is a separation between the insurers and the healthcare providers, mechanisms directly acting upon demand (such a as authorization of clinical services) emerge, whereas if the insurer shares the ownership with the healthcare providers, prevailing mechanisms act upon supply -control of the clinical practice and capitation payment. According to the interviewees, difficulties vary according to the mechanism, and they often refer to access to specialized care. On one side, *authorizations *delay care, especially in emergency transport and diagnostic testing: *"They lose time confirming if the patient is theirs or not [emergency transport], it can last from one minute to four hours just to get an answer. When they answer (...) it could last...from immediately, to six hours, to three days, according to the type of service requested*" (administrative, public primary care provider - urban subsidized network). The users from the rural area add costs in time and transportation, to do the paperwork and to obtain authorizations. The *limits to clinical practice *(medicines, tests and referrals to specialists) delay diagnosis and treatment. *Capitation payment *to healthcare providers has a direct negative impact on access by creating incentives to decrease the quantity and quality of services provided, and increasing waiting time and travel costs to capitated hospitals that become the first choice of insurers.

Two difficulties are added to subsidized enrollees of the urban area. First, the conflict between insurers and the local health authority in interpreting which services are included in the POS-S, as both want to avoid bearing the costs of the service. This results in more paperwork for patients and further delays in care (Table 4). The second is the way of services purchasing: the insurance companies split the process of care into various sub-processes to be purchased from different hospitals, requiring patients to go back and forth between various centers in order to resolve their health problems (Table 4). In addition, contracted healthcare providers frequently change, generating additional costs for patients due to errors in referral and the need to adapt them to the system (opening hours, documents, etc.).

The majority of public service professionals consider that barriers created by intermediation have made access to the continuum of healthcare services of subsidized enrollees worse than that of the uninsured who use the networks organized by the local health authority (Table 4).

### Barriers related to the network of healthcare providers

The barriers that predominantly emerge are those regarding the behavior of public healthcare providers in the subsidized regime, and the availability and geographic accessibility of the networks organized by insurers of both regimes. According to the informants, competition has triggered significant change in public hospitals, as they prioritize economic profit over patients' health needs (Table 5). It is considered a common practice for patients with profitable illnesses to be selected, and for patients who are uninsured, or lack the proper documentation to invoice the services, to be denied treatment. This is an especially critical issue in the urban area: *"Some diseases are not profitable; you see, for example, all of the chronic, internal medicine ones, because of the length of hospital stay and the cost of medicines. So people tend to provide care for easily resolvable diseases with short hospital stays and appropriate fees (...) and this goes against the population pyramid" *(manager, secondary care provider - rural subsidized network).

**Table 5:** Examples of quotations regarding "barriers related to the network of healthcare providers"

Category	Quotations
Changes in behavior of the public healthcare providers	*"The difference is that ten years ago we simply gave the patient what he needed, without asking where are you from, what *[insurance] *do you have or don't have, we'd just treated them and the State paid. Now we have to ask what he has, what's wrong, and who will pay or who to charge*." (manager, public secondary care provider - rural subsidized network).

Distance to specialized care services	*"Sometimes they just don't go, because on the one hand there's the bus ticket, which costs $6.000 *[2.5€] *roundtrip (...) and if they don't have it, they have to put up with the disease, because what can you do just with herbs?" *(user, subsidized regime-rural area)
	*"Many patents never get care because of that [geographic distance]. I have hypertensive patients who I've referred to internists and they've never gone (...) It's one zone in particular, and we're very far away" *(health professional primary healthcare provider - urban contributory network)

Causes: deficit in service supply	*"Sometimes the high technology hospital does not have a contract with certain institutions *[insurance companies of the subsidized regime], *and one finds oneself with the problem of having to send a patient somewhere, but not knowing where, you see? So the patient ends up staying in the emergency room because we can't find a place to send him." *(manager, subsidized secondary care provider - rural subsidized network)
	*"Bogotá is a very, very big city, with a gigantic deficit of beds. I think we're the only country in the world that calmly closes its two biggest *[public] *hospitals due to financial problems (...) (...) they should intervene and resolve the problem, but not close it (...)" *(manager, public primary care provider - urban subsidized network)

Waiting time	*"The waiting time is not good. I mean, I think that patients that make appointments that are relatively high priority are not getting them" *(health professional, public secondary care provider - urban contributory network

In-person and restricted appointment requirements	*"There are patients that sometimes get up at the crack of dawn, stand in line, aren't able to get an appointment, get tired of it, and a year passes, then two, and they don't get their specific antigen, even though they could be developing a serious illness*" (health professional, private secondary care provider - urban subsidized network)

In both regimes, the shortage in the supply of basic specialties and high technology medical services emerge, as well as limited geographic accessibility, both in the rural area and in the contributory urban network, which is aggravated by the low-income level of the population (Table 5). In the latter, problems in accessibility go all the way down to the first level of care. The causes of the shortage vary according to the regime: in the contributory regime, it is related to insufficient private investment and the closing of public hospitals. In the subsidized regime, informants point to insufficient public funding and to elements of managed competition: added administrative costs and incentives to close non-profitable services (internal medicine) or to insurers not contracting high technology services (Table 5).

Long waiting lists for specialized services, especially in emergency care, are the greatest organizational difficulty in both regimes and areas (Table 5). The informants relate it to insufficient supply and to the authorization requirements of insurance companies. In the subsidized regime, in-person and restricted appointment requirements, and the poor quality of care, are additional barriers to the access of specialized care (Table 5).

## Discussion

### Barriers in access to care and their interaction

**Figure 2:**
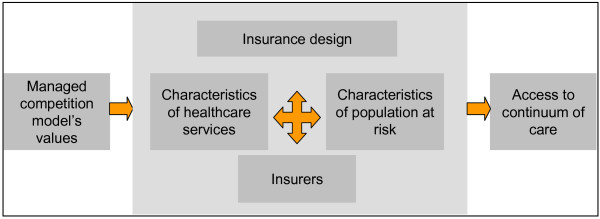
**Factors influencing access to the continuum of care based on categories emerging from the study**. Figure legend text: Source: authors.

Although the main objective of introducing managed competition in Colombia was to improve access to care [3], the results show several barriers of access to the continuum of care. These obstacles are not only due to factors usually taken into account in conceptual frameworks for access - services and population characteristics [47,50-52] - but also, and more importantly, to characteristics of the insurance design and to the presence of insurance companies. All are permeated with the values that introduced the managed competition model, that is the search for economic benefit or sustainability, which guides insurers and also healthcare providers' behavior. These values emerge in the discourse of the informants as a direct barrier to access or as a cause of other barriers (Figure 2).

These obstacles produce synergetic effects and increase the negative impact on the use of care, an aspect which is often omitted in quantitative research due to its difficult measurement [53]. For example, the insurers' authorization or in-person appointment requirements increase travel and time costs for patients, which are already high in networks whose services are hard to access geographically. Limits to first level of care tests and authorization requirements increase waiting times for specialized care, that are already long due to insufficient availability of services.

Many difficulties in access emerge in both insurance regimes and areas, but with remarkable differences. In the subsidized regime, financial obstacles predominate and are linked to a smaller specialized care services portfolio. In the contributory regime, structural deficiencies in the network prevail among the obstacles.

In the rural area, structural difficulties emerge more intensively due to insufficient public funds, a common problem in low-income countries with integrated public healthcare systems [54]. In the urban area there are strong barriers related to market functioning - changes of healthcare providers in the networks, conflict between paying entities, fragmentation of service provision across multiple providers, and the rejection of patients - coinciding with other studies [55].

### Causes of barriers to access

#### Segmented and insufficient insurance

The results show that subsidized insurance fails in facilitating access, not only due to the limited number of included services, coinciding with other studies [16,17], but because of the segmented design of the benefit package. Services that are excluded - usually in the initial phases of disease - are provided by networks funded by local authorities [3], which promotes conflict between health authorities and insurance companies to avoid assuming the funding. This, in turn, results in delays and sometimes in denial of care. In addition, there is an interruption in the continuum of care, which is aggravated by the classification of procedures by levels of care, and the contracting of different interventions for a healthcare process with different providers.

Due to insurance shortcomings, co-payments become some of the most significant barriers to access. This result, similar to other studies [56-59], contrasts with the decrease in the proportion of out-of-pocket expenses in total healthcare costs in Colombia, shown by official statistics [13]. This decrease seems to be concentrated mostly in those who are enrolled in the contributory regime and who have a higher income level [60].

#### Competition in insurance and in provision of services

The implications of competition in insurance management on access to healthcare services have been discussed on a theoretical level [61], and have generally focused on insurers' incentives to risk selection. In this study, however, difficulties emerge regarding how insurers influence the provision of care in aspects such as services availability along the continuum of care and its geographical or organizational accessibility; or the implementation of mechanisms to limit the use of services, such as authorization requirements or capitation payment. In the networks analyzed, that integrate insurance and provision of services, their influence is even greater, through the direct control of medical practice. The perceived notion that access along the continuum of care is more fluid for the uninsured highlights the significance of the barriers related to intermediation. The control exerted by insurers over the way in which patients receive healthcare has an extraordinary repercussion on equity as it will make access to health care vary among healthcare networks, and will increase the inequities already present in the system design [62].

These results are similar to those obtained by another study carried out in Colombia [39] and are also similar to the U.S. context. In the latter, systematic literature reviews show physician and enrollees' opinion against the use of managed care mechanisms, highlighting difficulties of access to drugs, tests and treatments [63,64].

Some authors argue that managed care techniques create dissatisfaction amongst physicians due to the loss of control and autonomy of clinical decision making [65]. However, the technique is not the problem in itself, but the goals that are set and the context in which it is used. GPs operating as gate-keepers, for instance, can be used to improve access to the health care system and relational continuity [66], but it can become an obstacle when it is used to control references to specialists, admissions and medical procedures, and thereby control costs [67]. Mechanisms such as capitation payment, that theoretically provide an incentive for efficiency may reduce the provision of needed services in environments such as developing countries with low services productivity.

On the other hand, the results show that competition in healthcare provision has meant a change in the values of public healthcare providers who now tend to prioritize economic sustainability - already described in other public health systems where the market was introduced into service provision [68] which incentives selection and, in some cases, rejection of patients.

These results also point towards the difficulty of introducing an effective regulatory system to correct market failures, that has been documented in developed as well as in a developing countries [69,70].

Therefore, the competition in insurance and provision of services in Colombia contributes to an increase in the negative impact of structural and organizational barriers common in most of the developing countries [54], through the increase of indirect costs - time and travel - and of delay in care due to the use of managed care mechanisms; as well as to the creation of new obstacles that do not appear in public models without competition, such as the denial of care in public facilities to uninsured people.

#### Characteristics of healthcare services

It is noteworthy that the shortage of specialized services and limited geographic accessibility emerge as barriers in the urban area, and for the insurance regime with larger resources (contributory). This seems to indicate that a greater benefit package does not translate into better access in economically disadvantaged areas. In fact, the Quality of Life Surveys show that the geographic barrier is an increasingly important reason not to seek healthcare services in the contributory regime [26]. These results reflect the difficult role of the health regulatory authority in guaranteeing that insurance companies offer networks with needed and geographically accessible services; and, consequently, how inappropriate it is for public authorities to devolve to the market planning decisions regarding investment in services. That there is an economic barrier also in the contributory regime is surprising, although data from the Quality of Life Surveys (Encuesta de Calidad y Vida - ECV) already showed that 10.6% of members of the contributory regime do not use services due to lack of money [26]. This barrier seems to be related more to the time and transportation cost because of geographic and organizational (authorization requirements) inaccessibility, than to co-payment.

### Limitations of the study

In the rural area, managers and administrative personnel of the EPS'S refused to participate and it was not possible to find a replacement case as it was the only insurance company operating in the area. The missing of these actors' opinions might mean that the information is not complete [71]. In the urban area, the only EPS of the contributory regime that agreed to participate was a non-profit insurance company, whose health services network was near to the area of study. These characteristics should be taken into account in the interpretation and transferral of results to other contexts [72]. With commercial EPS'S, it is reasonable to assume that obstacles would be greater.

## Conclusions

The origin of all of the problems of healthcare access in the Colombia public healthcare system can not be attributed to the managed competition model, but the results show how it has contributed to widen structural and organizational barriers of access to care, as well as to the creation of new ones that do not appear in public models without competition. Therefore, the results question the introduction of this type of model in low- and middle-income countries, as they show how extraordinarily difficult it is for a regulation scheme to effectively correct market failures, despite 16 years of regulatory effort. In these circumstances, the insurance companies and healthcare providers ultimately determine the conditions for the population's access to healthcare, and therefore, the level of equity of access in the healthcare system.

Moreover, this research has made possible the in-depth analysis of the factors influencing access to healthcare services that have rarely been considered in research in Colombia. It has also allowed some of the results obtained in other studies to be interpreted, such as the presence of economic and geographical barriers in the contributory regime [26]. This indicates the need for future surveys to distinguish between insurers' barriers and healthcare service barriers.

Both the health authority's inability to guarantee the provision of adequate care in rural and poor urban areas, and the persistence of barriers to health care, suggest the need for an integral reform rather than the partial measures (based on the assumption that improved regulation would ensure access) already adopted. A unique public system should be put into place, with integration of funding and health insurance, that is more equitable and easier to manage and regulate [73,74].

## Competing interests

The authors declare that they have no competing interests.

## Authors' contributions

IV and MLV were responsible for the study design and supervision of the field work and data analysis, and writing the article. IV conducted the field work and data analysis. ASM provided support for the field work and contribute to writing the article. JPU contributed to data analysis and writing of the article.

All authors participated in the revision and the fine-tuning of the final version of the article. The authors alone are responsible for the content of this paper.

## Pre-publication history

The pre-publication history for this paper can be accessed here:

http://www.biomedcentral.com/1472-6963/10/297/prepub
